# DWT-EMD Feature Level Fusion Based Approach over Multi and Single Channel EEG Signals for Seizure Detection

**DOI:** 10.3390/diagnostics12020324

**Published:** 2022-01-27

**Authors:** Gopal Chandra Jana, Anupam Agrawal, Prasant Kumar Pattnaik, Mangal Sain

**Affiliations:** 1 Interactive Technologies & Multimedia Research Lab, Department of Information Technology, CC-II, Indian Institute of Information Technology-Allahabad, Prayagraj 211015, India; go.gopal.ch.jana@gmail.com (G.C.J.); anupam@iiita.ac.in (A.A.); 2 School of Computer Engineering, Kalinga Institute of Industrial Technology, Bhubaneswar 751024, India; patnaikprasant@gmail.com; 3 Division of Computer Engineering, Dongseo University, 47 Jurye-Ro, Sasang-Gu, Busan 47011, Korea

**Keywords:** discrete wavelet transform, empirical mode decomposition, electroencephalogram, EEG classification, seizure detection

## Abstract

Brain Computer Interface technology enables a pathway for analyzing EEG signals for seizure detection. EEG signal decomposition, features extraction and machine learning techniques are more familiar in seizure detection. However, selecting decomposition technique and concatenation of their features for seizure detection is still in the state-of-the-art phase. This work proposes DWT-EMD Feature level Fusion-based seizure detection approach over multi and single channel EEG signals and studied the usability of discrete wavelet transform (DWT) and empirical mode decomposition (EMD) feature fusion with respect to individual DWT and EMD features over classifiers SVM, SVM with RBF kernel, decision tree and bagging classifier for seizure detection. All classifiers achieved an improved performance over DWT-EMD feature level fusion for two benchmark seizure detection EEG datasets. Detailed quantification results have been mentioned in the Results section.

## 1. Introduction

Electroencephalogram (EEG) signals decomposition and features extraction from the decomposed segments are popular approaches in seizure detection. A comprehensive context of EEG analysis and its applicability for automatic seizure detection has been discussed in [1], where authors have presented a survey by considering all possible papers that are used in the CHB-MIT EEG dataset. We studied and found that various decomposition approaches from the Discrete Wavelet Transform (DWT) and Empirical Mode Decomposition (EMD) family are used in seizure detection over EEG signals. Some approaches are studied and mentioned as follows to provide an overview of our contribution towards showing the usability of the proposed approach.

A discrete cosine transform (DCT) based approach [2] has been suggested in the context of EEG signals compression, and this provides us with a basic understanding of discrete transform even if it is applied over motor imagery EEG data. Similarly, a tunable-Q wavelet transforms (TQWT) and fractal dimensions-based approach [3] has been proposed for seizure detection. In the same context in [3], fractal dimensions features are extracted from all decomposed sub-bands; afterward, the features are fed into a support vector machine (SVM) with an RBF kernel. The authors of [3] claimed that their approach achieved a minimum accuracy of 98.50% and maximum accuracy of 100% over the University of Bonn single channel EEG dataset, but they have not tested their approach over a multi-channel EEG dataset. Another wavelet-based approach is named flexible analytic wavelet transform (FAWT) [4], which has been used for EEG signals decomposition to propose seizure detection approaches, where entropy-based features have extracted and been fed into SVM with an RBF kernel. The authors of [4] claimed that their approach achieved an accuracy of 94.41% over the Bern Barcelona database. In the literature [5,6,7,8], we observed that only DWT has been considered for EEG signals decomposition to propose a seizure detection approach. However, the impact of using DWT over the multi-channel EEG signals/data has not been addressed. However, the approach mentioned in [5] achieved an accuracy of 98% using a random forest classifier; the approach mentioned in [6] achieved an accuracy of 99.25% using an SVM classifier; and the approach mentioned in [7] achieved an accuracy of 100% with a GA-ANN classifier. The approaches mentioned in [5,6,7] have tested over the University of Bonn single channel EEG dataset only, but they have not used CHB-MIT or other multi-channel EEG dataset to test their proposed approach. On the other hand, the authors of [8] have considered the University of Bonn and CHB-MIT EEG datasets (i.e., single and multi channel EEG dataset) to test their approach and claimed that their approach with SVM achieved an accuracy of 89.03% over CHB-MIT and 97.67% over Bonn.

Similarly, several studies have been conducted for seizure detection by considering only EMD-based decomposition approaches. We observed in most of the studies [9,10,11,12] that the authors have used EMD as baseline technique for EEG signals decomposition and, after that, they have extracted several features from its derivative (namely Intrinsic Mode Functions, i.e., IMFs). The authors of [9] claimed that EMD with SVM, KNN, naive Bayes and logistic regression classifiers achieved an accuracy of 94.56%, 95.63%, 96.8% and 96.25%, respectively, and using ensemble EMD with SVM, KNN, naive Bayes and logistic regression obtained an accuracy 96.06%, 97%, 97% and 96.25%, respectively. In [10], the authors claimed that their approach achieved an accuracy, sensitivity and specificity of 92.9%, 94.3% and 91.5%, respectively, over the CHB-MIT dataset. The authors of [11] mentioned that their approach achieved an accuracy of 90% (for 50 pairs of signals) and 82.53% (for 750 pairs of signals) over a Bern Barcelona database. Similarly, in [12], the authors mentioned that their approach achieved an average accuracy of 99.3% to 100% for the SVM-based classifier over the Bonn dataset.

In another study, an extended version of EMD named Multivariate Empirical Mode Decomposition [13] has been proposed for seizure detection. However, the authors have not studied the usability of considering EMD over multi-channel EEG signals/data. However, the proposed approach in [13] achieved an acceptable classification (i.e., detection) accuracy of 87.2% over the University of Bonn single channel EEG dataset.

On the other hand, in [14], the authors have proposed a seizure detection approach based on DWT analysis of the dominant IMFs resulting from the EMD of the EEG signals. The authors of [14] claimed that their approach achieved an average accuracy of 100% over the Bonn dataset, but they have not tested their approach over the multi-channel EEG dataset. Instead of using DWT and EMD sequentially, the authors of [15] have used DWT and EMD individually for seizure detection and achieved an average accuracy between 92.27% to 97.18% over the CHB-MIT multi-channel EEG dataset. With the concept of wavelet, a Fourier–Bessel series expansion-based empirical wavelet transform approach [16] has been proposed for the elimination of ocular artifacts from EEG single, and the authors claimed that their approach achieved a mean absolute error of 0.029 for alpha rhythm. In addition to [14], another sequential use of DWT and EMD has been proposed by the authors of [17] for seizure detection and achieved a highest accuracy of 100% for the use of EMD and DWT, respectively, over the single channel EEG dataset; moreover, they have not tested their approach over the multi-channel EEG dataset. The study in [18] has been showed the usability of DWT and EMD for EEG channel selection for seizure detection over the CHB-MIT EEG dataset, and the authors mentioned that their approach achieved 95.00% accuracy for the use of all EEG channels and 97.50% for the use of only two EEG channels. Similarly to DWT a tunable-Q wavelet transform-based approach, Ref. [19] has been proposed seizure detection, and the authors mentioned that their approach achieved an accuracy of 99% over the Bonn EEG dataset, but they have not tested their approach over a multi-channel EEG dataset. Not only in seizure detection has the DWT and EMD mixture models been used, in [20] a probability based Generalized Mixture Distribution Model has also been used for emotion classification by using EEG signals, and the authors claimed that their approach achieved an accuracy of 89%.

After this literature study, we observed that most of the studies used both DWT and EMD decomposition techniques individually or sequentially to propose seizure detection approach. However, there is a scope of extracting the DWT and EMD coefficient features parallelly at a time from an EEG segment, and it is possible to extract statistical features from the coefficients and to concatenate them for seizure detection. Thus, in the same context, we motivated to work on to concatenate DWT coefficient-based feature matrix and EMD IMF-based feature matrix.

Moreover, a conclusive performance analysis has been performed as to whether our proposed approach has any usability over single and multi-channel EEG signals to detect seizure (more specifically, ictal and non-ictal).

### Our Contributions

(1) We have proposed a seizure detection approach based on the concatenation of DWT coefficient-based feature matrix and EMD IMF-based feature matrix.
(2) We have tested our proposed approach over the single and multi-channel EEG datasets to provide a conclusive analysis with four classifiers with respect to DWT and EMD approaches individually.
(3) This study investigates and suggest the prominent usability DWT-EMD-based features concatenation over the multi-channel EEG signals with respect to usability over single channel EEG signals.

The rest of the manuscript is organized as follows: In Section 2, materials and methods (including illustration of the proposed approach) have been described. We have shown the results and the discussion (including comparison with existing schemes) of this paper in Section 3. Then, conclusions and future scope are mentioned in Section 4.

## 2. Materials and Methods

This section describes the materials and methods used in this study. Section 2.1 describes experimental data and baseline methods, and Section 2.2 illustrates the proposed approach.

### 2.1. Experimental Data and Baseline Methods

This subsection describes experimental data and baseline methods. Section 2.1.1, describes experimental data, and Section 2.1.2 describes baseline methods as follows.

#### 2.1.1. Experimental Datasets

In this study, we have used two benchmark EEG datasets, namely dataset1 and dataset2. A brief description of the dataset1 and dataset2 is mentioned in the following.

Dataset1: In this study, we have used the CHB-MIT scalp EEG dataset [21,22] as an experimental dataset1. In dataset1, we have considered ten cases (i.e., chb01 to chb10) out of twenty-three cases. The considered ten cases have recordings of 23 EEG channels. With reference to a study presented in [23], we have considered the channels F3-C3, C3-P3, F4-C4 and C4-P4, which reflect actable seizure detection performance in their study. Then, we have utilized 57 ictal files that vary with the ictal time period and 57 non-seizure files for data balancing. Non-ictal data contain 60 s of data in each file.

Dataset2: We have used the University of Bonn EEG dataset [24,25] as a second experimental dataset, i.e., Dataset2. It has five zip files named set A, B, C, D and E, and each zip file contains 100 text filles and each text file possesses 4096 samples of one EEG time series. Each recording has a duration of 23.6 s with a sampling frequency of 173.6 Hz. In this study, we have considered Set A (healthy patients, i.e., having non-ictal data) and Set E (seizure patients, i.e., having ictal data).

#### 2.1.2. Baseline Methods

In this subsection, we have mentioned all baseline methods that were employed in this study.

(1) Preprocessing: EEG recordings sometime have a few noisy segments due to loosened electrode placement, subject eye blinking and muscle activities. Thus, there is a requirement of basic preprocessing. In this study, we have applied a Butterworth [26,27,28] second-order band pass filter in the frequency range of 0.5–70 Hz for basic preprocessing.
(2) Signal Decomposition using DWT: EEG signals are non-stationary [29]. In nature, this means that its behavior varies with respect to time. Discrete wavelet transforms (DWT) [30,31,32] decompose input signals and produces a set of characteristic signals in the form of approximation coefficients and detail coefficients. An input signal passes into a series of filters to estimate DWT. Consider an input signal *‘S’* passing into a series of filters to estimate its DWT. Firstly, the signals are passed into a low-pass filter with an impulse response, say ‘*G’*. Equation (1) expresses this mathematically.

(1) $$y[n]=(S*G)[n]=\sum_{k=-\infty }^{\infty }S[k]G[n-k]$$

Moreover, the input signals are simultaneously decomposed by using a high-pass filter. In DWT, the low-pass filter produces outputs as an approximation coefficient, and the high-pass filter produces outputs as a detail coefficient.

**Figure 1:**
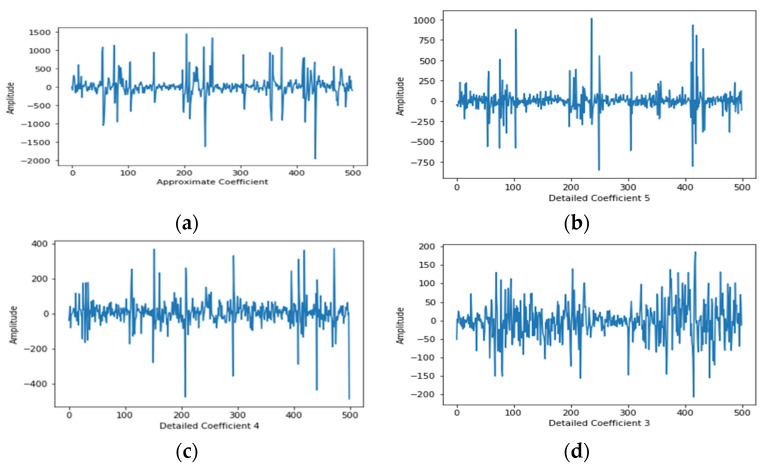

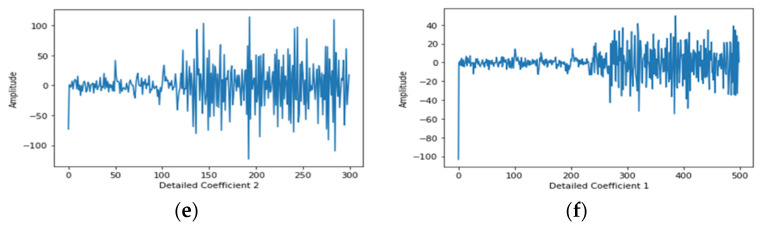
Shows all coefficients of five levels of DWT over experimental EEG signals. (**a**) shows the approximate coefficient, and (**b**–**f**) shows other detailed coefficients of DWT level 5.

In this proposed approach, we have used 5 levels of decomposition (after performing several test cases of choosing levels of decomposition) with ‘Haar wavelet’ [33] as the mother wavelet function. The illustrative output (i.e., approximation and detail coefficients) of experimental EEG signals is shown in Figure 1a–f.

**Figure 2:**
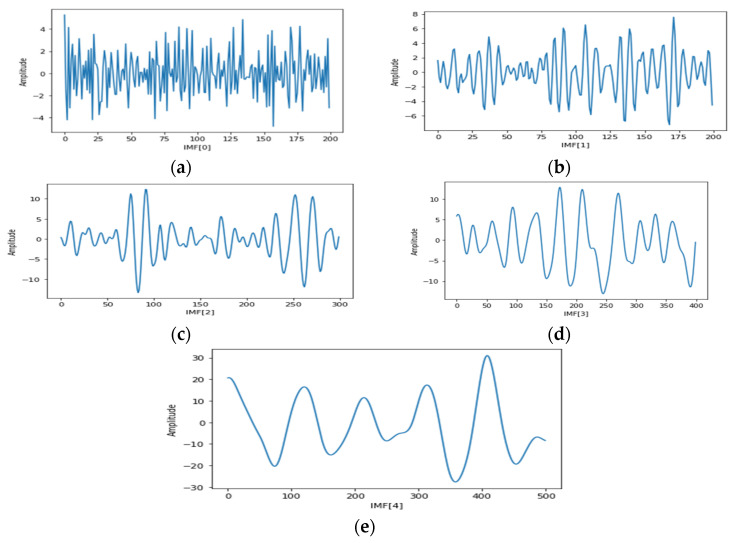
Shows five IMFs of EMD applied on chb01_01 of Dataset2. IMF0, IMF1, IMF2, IMF3 and IMF4 are shown in (**a**–**e**) accordingly.

(3) Signal Decomposition using EMD: Empirical mode decomposition (EMD) is a more popular technique for non-stationary signals decomposition [34,35,36]. EMD decomposes its input signals into different intrinsic mode functions (IMFs). IMFs follow two main properties [35]: (a) the count of local minima and maxima varies as a maximum by one and (b) has a mean value of zero. Algorithmic and conceptual details have been reported in [34,35]. In this experiment, the EMD technique has been applied on input EEG signals from the both datasets, and a few sample outputs are plotted and shown in Figure 2a–e.

(4) Statistical Feature extraction: In the feature extraction process, seven statistical features have been extracted from DWT coefficients, and six features have been extracted from IMFs of EMD. The extracted features from DWT coefficients are mean (Equation (2)); variance (Equation (3)); standard deviation (Equation (4)); curve length (Equation (6)); skewness (Equation (8)); kurtosis (Equation (9)); and minima (Equation (7)). On the other hand, variance; Root Mean Square (RMS) (Equation (5)); standard deviation; curve length; skewness; and kurtosis features have been extracted from IMFs of EMD. The formula of each considered features is presented in Table 1.

(5) DWT-EMD Features Level Fusion: Feature concatenation has been performed individually for both experimental datasets. The detailed process is described as follows.

For Dataset1, it has ictal and non-ictal EEG recordings. We have considered ictal recordings as per the time stamp contained in the dataset description. As ictal periods vary subject to subject, the feature matrix (after DWT, EMD and statistical feature extraction), hence, also vary for the ictal periods after feature concatenation. For non-ictal periods, we have considered 60 s recordings from non-ictal files. Dataset1 has EEG recordings with 256 Hz sampling frequency. Thus, the number of datapoints taken to ensure feature extraction is 60 × 256 = 15,360. The number of channels considered is 4, which resulted in the shape of (15,360, 4) (initial data matrix before DWT, EMD and feature extraction). Currently, DWT (Haar, level-5) has been applied to each channel. After DWT, for each channel, we received 6 coefficients (one approximate coefficient and five detailed coefficients). Thus, we now obtain 24 (4 × 6) DWT coefficients features from the four channels. We have currently extracted 7 statistical features (mentioned in the features extraction section) from each DWT coefficients (a total of 24). Thus, we have 168 (24 × 7) statistical features for each seizure (ictal) and non-seizure (non-ictal) file. We have considered 58 seizures (ictal) and non-seizure (non-ictal) files, and the resultant feature matrix (after DWT and statistical feature extraction) has a shape of (58, 168) for the both seizure and non-seizure.

Similarly, EMD has been applied on 4 channels individually, and 5 IMFs have been considered from each channel for further feature processing (i.e., we have 20 IMFs from 4 channels). Then, six statistical features were extracted from each IMFs. Thus, we have 120 (4 × 5 × 6) statistical features for one seizure (ictal) file and for non-seizure (non-ictal) files (we have considered 58 seizure and non-seizure files, respectively). Finally, for EMD, we have a feature matrix of shape of (58, 120) for considered seizure segments and the same for the considered non-seizure segments. Now, concatenation of two feature matrixes (DWT coefficient-based statistical feature matrix and EMD IMF-based statistical feature matrix) has been performed for the both seizure and non-seizure feature representations.

For Dataset 2, we considered two sets (Set-A and Set-E) as experimental dataset 2, and further feature processing has been carried out. Set-A and Set-E each contain 100 files, with each file having 4096 EEG data points. We have split 4096 points into 8 files possessing 512 points each. Thus, for seizure and non-seizure, we have 800 (100 × 8) data segments with 512 EEG data points. Currently, we applied DWT (Haar, level-5) over each data segment (having 512 EEG data points) and estimated 6 DWT coefficients (one approximate coefficient and five detailed coefficients) features. After, 7 statistical features have been extracted from each DWT coefficients for all EEG data segments. Thus, we have 42 (6 × 7) features based on DWT and statistical features. Therefore, after DWT and statistical feature extraction, we have a feature matrix shape of 800 × 42. Simultaneously, we have applied EMD over all data segments, and 5 IMFs have been extracted from each data segments. Then, 6 statistical features have been extracted from each IMFs for all EEG data segments. Thus, we obtain 30 (5 × 6) features based on EMD and statistical features. Therefore, after EMD and statistical feature extraction, we have a feature matrix shape of 800 × 30. The same process has been followed for seizure (Set-E) and non-seizure (Set-A) EEG data.

Concatenation of two feature matrices (DWT coefficient-based statistical feature matrix and EMD IMF-based statistical feature matrix) has been conducted for both seizure and non-seizure feature representations.

(6) Classifiers: In this experiment, we used four classifiers, namely support vector machine (SVM) without kernel and with RBF kernel; decision tree (DT); and a bagging classifier to estimate seizure detection (i.e., ictal and non-ictal classification) performance over DWT and EMD-based statistical features. The baseline of the considered classifiers is mentioned as follows.

SVM Classifier: SVM serves as a classifier and regression model on the basis of optimal hyperplane [41,42]. It separates the features in linearly or nonlinearly distinguishable patterns [43]. Here, we have used SVM without kernel and SVM with RBF kernel (C = 100, default gamma value) as a classifier. Algorithmic details of SVM and its application can be found in [44].

Decision Tree Classifier: This is one of the more popular non-parametric approaches for multistage decision making [45]. The main concern of multistage decision making is to divide complex decisions into several nodes of simpler decisions [45]. In a decision tree, features are represented by nodes, and categorical outcomes are represented by leaves [46]. A decision tree uses the Gini index and entropy as measures for splitting a node [45,46]. More extensive explanations of the Gini index and entropy measurements for splitting measures appear in [46]. In this experiment, we have used the criterion (default) as Gini (for Gini impurity) and Entropy (for the information gain). The maximum depth of the tree is 4 (i.e., max_depth = 4).

Bagging Classifier: This is a meta-ensemble approach that takes random subsets as inputs and predicts the outcome after combining the results from different classifiers [47]. The classifiers are run in parallel, and they learn independently. It helps in reducing variances of data and, therefore, reduces overfitting [48,49]. More extensive explanations of the bagging classifier are mentioned in [47]. In this experiment, the bagging classifier has been configured with a decision tree as a base estimator (i.e., base_estimato = dt) in order to fit on random subsets of the dataset. After several test cases, we have considered 300 base estimators in the ensemble (i.e., n_estimators = 300).

### 2.2. Illustration of Proposed Approach

**Figure 3:**
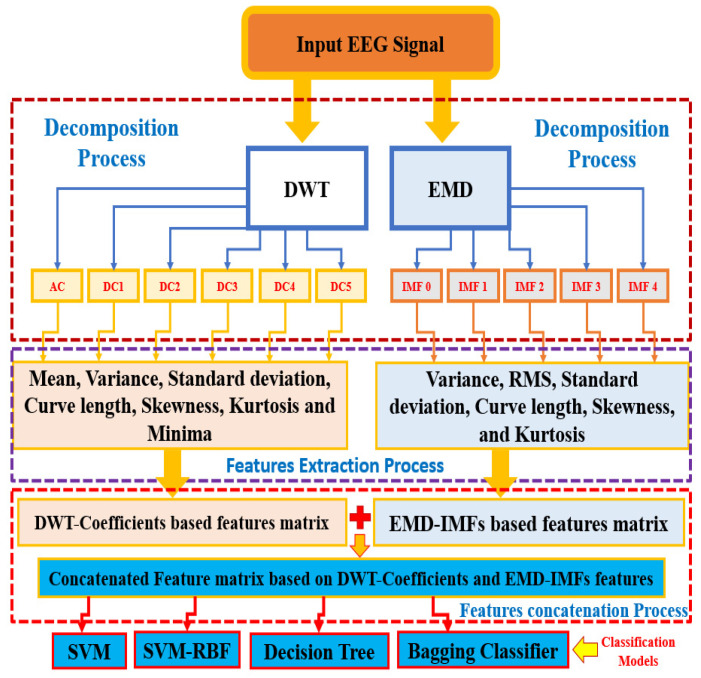
Illustrative diagram of the proposed approach.

Proposed DWT-EMD features concatenation-based seizure detection approaches use supervised learning for discriminating ictal and non-ictal EEG signals. An illustrative diagram of the proposed approach is shown in Figure 3. The input EEG signal mentioned in Figure 3 is considered after applying a Butterworth filter. Detail descriptions of each entity of the Figure 3 are mentioned in Section 2.

## 3. Results and Discussion

Performance evaluation has been performed on three cases over two (multi and single-channel) benchmark EEG datasets (Dataset-1 and 2) in order to visualize the usability of proposed DWT-EMD features concatenation-based seizure detection approach. Three different cases have been taken into consideration in this study. Thus, the considered cases are as follows.

Case 1: Input EEG signals are preprocessed by using the Butterworth filter; then, DWT (Haar and level-5) is applied. Then, seven features have been extracted from each resultant DWT coefficient. Finally, the DWT coefficient-based statistical features matrix is fed into the considered classifiers in order to estimate seizure detection (i.e., ictal and non-ictal classification) performance.

Case 2: Input EEG signals are preprocessed using the Butterworth filter; then, EMD is applied to extract 5 IMFs. Then, six features have been extracted from each IMFs. Finally, the EMD IMF-based statistical features matrix is fed into the considered classifiers in order to estimate seizure detection (i.e., ictal and non-ictal classification) performance.

Case 3: In case three, we have considered feature matrices of case 1 and 2 for features concatenation, i.e., the DWT coefficient-based feature matrix and EMD IMF-based feature matrix have been concatenated; then, the final DWT and EMD-based feature matrices are fed into the considered classifiers for estimating seizure detection (i.e., ictal and non-ictal classification) performance.

Seizure detection performance of the different classifiers over different cases has been evaluated by using the following measures: Accuracy score, F1 score and Matthews correlation coefficient (MCC). All considered performance evaluation parameters are very standard; thus, we are not describing them by using formulas. The results of the considered cases are discussed in Section 3.1.

We have used Google Colab Python environment for implementing the source code of this experiment and used the improved results achieved with the mentioned hyperparameter only.

### 3.1. Results

In this subsection, we have presented the experimental results of this study by using different subsections as follows.

#### 3.1.1. Performance under Case-1

In case-1, the statistical feature matrix is based on DWT coefficients. The estimated best performance of the considered classifiers over both datasets under case-1 is provided in Table 2 and Table 3. Table 2 shows the performance of Dataset-1 (multi-channel), where we can observe that all classifiers achieved an accuracy between 80 and 82 percent, but MCC has noticeable variance, and very few variances are observed for a few of classifiers, which is not acceptable in any binary classification task. This advises us to work on the feature matrix to improve performance.

On the other hand, Table 3 shows the estimated performance of all classifiers over Dataset-2 (single channel) under case-1. From Table 3, we can observe that all classifiers achieved higher performance in comparison to performance achieved on Dataset-1 under case-1.

#### 3.1.2. Performance over Case-2

In case-2, the statistical feature matrix is based on EMD IMFs. Estimated best performance of the considered classifiers over both datasets under case-2 is provided in Table 4 and Table 5. Table 4 shows the performance of Dataset-1 (multi-channel), where we can observe that all classifiers achieved an accuracy with a high percentage between 80 and 91, but MCC percentage is only high for the bagging classifier. Thus, the overall performance is not good for this binary classification with respect to MCC. This advises us to work on the feature matrix in order to improve the performance of all classifiers.

On the other hand, Table 5 shows that all classifiers achieved an acceptable performance with high accuracy, F1 score and MCC over the Dataset-2 under case-2.

#### 3.1.3. Performance over Case-3

In case-3, the statistical feature matrix is based on DWT coefficients and EMD IMFs. The estimated best performance of the considered classifiers over both datasets under case-3 is provided in Table 6 and Table 7. Table 6 shows the performance over Dataset-1 (multi-channel) under case-3, where we can observe that all classifiers achieved improved performances in comparison to the performances shown in Table 2 and Table 4. In case-3, all classifiers achieved high accuracies above 91 percent, F1 score above 90 percent and MCC above 82 percent. The results of case-3 under Dataset-1 advise us to consider the concatenated DWT-EMD feature matrix in order to improve seizure detection performance over multi-channel EEG signals. Therefore, the proposed approach has true usability for seizure detection over multi-channel EEG data.

On the other hand, Table 7 shows similar performance as those observed in Table 3 and Table 5 over Dataset-2 (single channel) under case-1 and case-2, respectively.

The performances mentioned in Table 3, Table 5 and Table 7 advise us of the following: The proposed DWT-EMD feature concatenation approach is not very beneficial for improving seizure detection performance over single-channel EEG signals. However, Table 7 shows improved performance in comparison to the performances shown in Table 3 and Table 5. Therefore, the proposed approach also has some usability for seizure detection over single-channel EEG data.

### 3.2. Comparison with Existing Schemes

In this study, we have compared ictal and non-ictal classification performances of our proposed approach (seizure detection using DWT coefficients and EMD IMF-based EEG features concatenation) with suitable existing state-of-the-art approaches over the same experimental datasets. Most of the existing work used Dataset-2 (University of Bonn Single channel EEG dataset) for analyzing the performance of their proposed approach. With the help of Table 2, Table 3, Table 4, Table 5, Table 6 and Table 7, we have shown a detail experimental analysis over two datasets (Dataset-1 (multichannel) and Dataset-2 (single channel)) by considering four classifiers under three cases. This comparison indicates the usability of DWT-coefficients and EMD-IMF-based EEG features concatenation in seizure detection over multi and single-channel EEG signals.

On the other hand, comparisons are presented in Table 8 to understand the relevance of our proposed work on existing similar state-of-the art approaches. Several schemes have been suggested by using DWT and EMD decomposition techniques for seizure detection over the same datasets. Vipin Gupta et al. [4] proposed flexible analytic wavelet transform (FAWT)-based seizure detection approach and achieved accuracies of 94.41% and 93.80% and MCC of 89% and 88% using LS-SVM and KNN, respectively, over Dataset-2 (single channel EEG dataset). On the other hand, our proposed approach with four classifiers achieved an accuracy between 99.37 and 100% and MCC between 98.75 and 100% over Dataset-2. Similarly, Anurag Nishad et al. [19] reported a seizure detection approach based on tunable-Q wavelet transform (TQWT), and the authors have claimed that their approach achieved an accuracy of 99% using random forest classifiers over the features taken from Dataset-2. On the other hand, our proposed approach achieved higher performance (than reported in [19]) by all four classifiers over Dataset-2. Another seizure detection scheme has been proposed by Mehdi Omidvar et al. [7], where authors have used several features from DWT coefficients to perform the detection process. In [7], the authors have claimed that classifiers ANN and SVM both achieved 100% accuracy over Dataset-2. To compare with this, if we observe the performance (mentioned in Table 7 and Table 8) of the considered classifiers of our proposed approach, it is observed that each classifier achieved an accuracy between 99.37 and 100%, F1 Score between 99.38 and 100% and MCC between 98.75 to 100% over Dataset-2.

In another published work, a similar approach has been reported by Duo Chen et al. [8] where several statistical and morphological features have been considered from DWT coefficients for seizure detection over Dataset-1 (multi-channel) and Dataset-2 (single channel). In [8], the authors have claimed that SVM with RBF kernel achieved an overall maximum accuracy of 92.30% and 99.33% over Dataset-1 and Dataset-2, respectively. On the other hand, our proposed approach achieved the highest accuracy and F1 score and MCC of 94.28%, 94.73% and 89.11%, respectively, over Dataset-1. Moreover, our proposed approach achieved better performance in terms of accuracy between 99.37 and 100%, F1 Score between 99.38 and 100% and MCC between 98.75 and 100% over the Dataset-2. Other than DWT or any wavelet-based decomposition techniques, EMD has been used by many researchers for seizure detection. An EMD-based seizure detection approach has been suggested by Kaleem Muhammad et al. [10], and the authors have claimed that the SVM classifier achieved an accuracy of 92.91% over projection coefficient value features from Dataset-1. On the other hand, our proposed approach with the bagging classifier achieved an accuracy of 94.28%, F1 score of 94.73% and MCC of 89.11% over Dataset-1.

In another proposed study by Wijayanto Inung et al. [12], EMD and coarse-grained (CG) EMDs have been applied to seizure detection over Dataset-2. In [12], the authors have claimed that KNN, RF and SVM classifiers achieved an accuracy of 99%, 99% and 100%, respectively, over Dataset-2. On the same dataset (i.e., Dataset-2), our proposed approach with classifiers SVM, SVM-RBF, decision tree and bagging classifier achieved an accuracy of 99.37%, 100%, 99.58% and 100%, respectively. Another seizure detection approach has been proposed by Asmat Zahra et al. [13], where MEMD using Hilbert transform has been applied for feature extraction. In [13], the authors have claimed that the proposed approach with ANN achieved an accuracy of 87.20% over Dataset-2. On the other hand, our proposed approach achieved the lowest accuracy of 99.37% by SVM and the highest accuracy of 100% by bagging classifier over Dataset-2. Moreover, we have studied and considered the work proposed by C. Shahnaz et al. [14], Shaik. Jakeer Hussain et al. [15] and Marzhan Bekbalanova et al. [17] for comparison (in terms of seizure detection performance) with our work. In [14], the authors have used EMD-wavelet analysis over Dataset-2 and extracted variance, skewness and kurtosis features for seizure detection. The authors of [14] mentioned that the proposed approach with KNN achieved an accuracy of 100% over Dataset-2 (which is a single channel), but the multi-channel EEG dataset has not been tested using their proposed approach. In our proposed work, we have tested four classifiers over Dataset-1 (multi-channel) and Dataset-2 (single channel), and we achieved the highest accuracy of 94.28% by bagging classifier and 100% SVM-RBF and bagging classifier over the two datasets, respectively. In [15], another seizure detection approach has been proposed by using DWT and EMD decomposition techniques, where the mean weighted frequency feature has been extracted individually from DWT coefficients and EMD IMFs. The authors of [15] have mentioned that their proposed approach with ANN achieved an accuracy of 91.85% (using DWT) and 92.27% (using EMD) for multi subject over Dataset-1. On the other hand, our proposed approach achieved an accuracy of 94.28% with a good MCC of 89.11% by bagging classifier over Dataset-1. In another study, Marzhan Bekbalanova et al. [17] utilized DWT and EMD for seizure detection over Dataset-2. In [17], the authors have mentioned that they have extracted features (mean, variance, skewness and kurtosis) from DWT coefficients and EMD IMFs individually. The authors of [17] have used SVN, KNN and decision tree classifiers and achieved an accuracy of 99%, 97.5% and 100% over DWT-based features and 100%, 100% and 96.25% over EMD-based features. On the other hand, our proposed approach achieved an accuracy of 99.37% by SVM, 100% by SVM-RBF, 99.58% by DT and 100% by bagging classifier over Dataset-2. Therefore, from Table 8, it has been observed that most of the studies [4,7,8,12,13,14,19] used DWT or EMD or DWT with EMD over Dataset-2 (single-channel EEG dataset) and reported seizure detection performance.

Moreover, a few of the studies [8,10,15] used multi-channel EEG data, but DWT coefficient-based features and EMD-IMF-based features have not been concatenated for both Dataset-1 and Dataset-2. In our study, we have used both single channel EEG datasets (Dataset-2) as well as multi-channel EEG dataset (Dataset-1) to understand the usability of our proposed approach. We observed that our proposed approach helps to improve seizure detection accuracy over the multi-channel EEG dataset and over the single-channel dataset. Moreover, the use of DWT or EMD individually over signal channel EEG data produces good and acceptable seizure detection accuracies. Thus, there is no compulsion in performing concatenation of DWT-coefficient features and EMD-IMFs features for seizure detection over single-channel EEG data if the data are already producing high detection performances when using only DWT or EMD techniques. However, our proposed approach achieved improved performance over Dataset-2. In the case of the multi-channel EEG dataset, it is better to consider our proposed approach because it demonstrates improved performance for all four classifiers with respect to the existing state-of-the art approaches over Dataset-1. Therefore, from Table 8, we can understand the usability of our proposed approach for seizure detection over single and multi-channel EEG signals.

However, this study has the following limitations: (1) The EMD mode mixing problem has not considered; and (2) the proposed approach has less impact on improving the results over single-channel EEG data.

## 4. Conclusions with Feature Scope

In this paper, an approach based on DWT-coefficient features and EMD-IMFs feature concatenation has been proposed for ictal and non-ictal classification over single and multi-channel EEG signals. A DWT-coefficients and EMD-IMF-feature-based concatenated input features matrix has been constructed by concatenation of the features extracted from six DWT-coefficients and five EMD-IMFs. Three cases have been taken into account in order to understand the usability of the proposed approach. Four classifiers (SVM, SVM-RBF, decision tree and bagging classifier) have been used in order to check uniformity in classification performance over the concatenated input features matrix. The performance of the proposed approach has shown improved and better classification performance than existing suitable approaches. Specifically, the performance of the proposed approach is as follows: SVM achieved 91.42% accuracy, 91.42% F1 Score and 83.00% MCC; SVM-RBF achieved 91.42% accuracy, 90.32% F1 Score and 82.78% MCC; decision tree achieved 91.42% accuracy, 92.30% F1 Score and 84.01% MCC; bagging classifier achieved 94.28% accuracy, 94.73% F1 Score and 89.11% MCC for Dataset-1 (Multi-Channel) and for Dataset-2 (Single-Channel); SVM achieved 99.37% accuracy, 99.38% F1 Score and 98.75% MCC; SVM-RBF achieved 100% accuracy, 100% F1 Score and 100% MCC; decision tree achieved 99.58% accuracy, 99.56% F1 Score and 99.16% MCC; and bagging classifier achieved 100% accuracy, 100% F1 Score and 100% MCC. However, from Table 2, Table 4 and Table 6, we can say the proposed approach is more suitable for long-term multi-channel EEG data. A detailed comparison with state-of-the-art approaches has been shown in Table 8. The comparison shows that the proposed approach has effective usability in seizure detection (more specifically, ictal and non-ictal classification) over single and multi-channel EEG signals. This proposed approach can be utilized for motor imagery, autisms, Alzheimer’s and schizophrenia detection using EEG signals.

## Figures and Tables

**Table 1:** Mathematical formula of considered features.

Considered Features	Mathematical Representation	Equation No.
Mean (μ)	$\mu =\frac{1}{n}\sum_{i=1}^{n}x_{i}$	(2)
	In Equation (2), mean is denoted as ′μ′.′n′ is total number of samples, $x_{i}\in \left\{x_{1},x_{2},x_{3},\ldots x_{n}\right\}$ denoting EEG time series sample points. ‘*i*’ is an integer number that belongs to 1 to *n*. More related details can be found in [17,37].	
$\mathrm{Variance}\;\left(\sigma^{2}\right)$	$\sigma^{2}=\frac{1}{n}\sum_{i=1}^{n}{\left(x_{i}-\mu \right)}^{2}$	(3)
	In Equation (3), variance is denoted as $\sigma^{2}$. ′n′ is total number of samples. $x_{i}\in \left\{x_{1},x_{2},x_{3},\ldots x_{n}\right\}$ denoting EEG time series sample points. ‘*μ’* is the estimated mean (refer Equation (2)) of the considered samples. ‘*i*’ is an integer number that belongs to 1 to n. More related details can be found in [17,37].	
Standard deviation (σ)	$\sigma =\sqrt{\frac{\sum_{i}^{n}{\left(x_{i}-\mu \right)}^{2}}{n}}$	(4)
	In Equation (4), standard deviation is denoted as σ. ′n′ is total number of samples. $x_{i}\in \left\{x_{1},x_{2},x_{3},\ldots x_{n}\right\}$ denoting EEG time series sample points. ‘*μ’* is the estimated Mean (refer Equation (2)) of the considered samples. ‘*i*’ is an integer number that belongs to 1 to *n*. More related details can be found in [37].	
Root Mean Square (RMS)	$RMS=\sqrt{\frac{1}{n}\sum_{i=1}^{n}x_{i}^{2}}$	(5)
	In Equation (5), Root Mean Square is denoted as RMS. ′n′ is the total number of samples. $x_{i}\in \left\{x_{1},x_{2},x_{3},\ldots x_{n}\right\}$ denoting EEG time series sample points. ‘*i*’ is an integer number that belongs to 1 to *n*. More related details can be found in [38].	
Curve length	$CL(n)=\log(\sum_{i=2}^{n}\left|x_{i}-x_{i-1}\right|)$	(6)
	In Equation (6), curve length is denoted as CL(n). ′n′ is total number of samples. $x_{i}\in \left\{x_{1},x_{2},x_{3},\ldots x_{n}\right\}$ denoting EEG time series sample points. ‘*i*’ is an integer number that belongs to 2 to *n*. More related details can be found in [39].	
Minima	$A_{\min}=\;|A_{n}|\;,\;if\;|A_{n}|\langle |A_{n+1}|and|A_{n}\langle A_{n-1}|$	(7)
	In Equation (7), Minima denoted as $A_{min}$. A implies amplitude, and *’n’* is the total number of samples. More related details can be found in [40].	
Skewness	$sk=\frac{1}{n}{\sum_{i=1}^{n}\left(\frac{x_{i}-\mu }{\sigma }\right)}^{3}$	(8)
	In Equation (8), Skewness is denoted as ′sk′, *σ* is the standard deviation (refer Equation (4)) of the considered samples, ’*n*’ is total number of samples and $x_{i}\in \left\{x_{1},x_{2},x_{3},\ldots x_{n}\right\}$ denoting EEG time series sample points. ‘*μ’* is the mean (refer Equation (2)) of the considered samples. ‘*i*’ is an integer number that belongs to 1 to *n*. More related details can be found in [17].	
Kurtosis	$Ku=\frac{1}{n}{\sum_{i=1}^{n}\left(\frac{x_{i}-\mu }{\sigma }\right)}^{4}$	(9)
	In Equation (9), Kurtosis is denoted as ′Ku′. *σ* is the standard deviation (refer Equation (4)) of the considered samples. ’*n*’ is total no. of samples, $x_{i}\in \left\{x_{1},x_{2},x_{3},\ldots x_{n}\right\}$ denoting EEG time series sample points. ‘*μ’* is the mean (refer Equation (2)) of the considered samples. ‘*i*’ is an integer number that belongs to 1 to *n*. More related details can be found in [17].	

**Table 2:** Estimated performance over Dataset-1 under case-1.

Classifier Used	Best Performance with Hyperparameters	Accuracy *	F1 Score *	MCC *
SVM + RBF	C = 100, kernel = ‘rbf’	82.85	64.28	85.71
SVM	default	82.85	83.33	70.71
Decision Tree	criterion = ‘gini’, max_depth = 4	80.00	55.55	11.78
Bagging Classifier	base_estimator = dt, n_estimators = 300, max_samples = 0.5	80.00	82.92	58.92

* Unit of measurement considered as percentage.

**Table 3:** Estimated performance over Dataset-2 under case-1.

Classifier Used	Best Performance with Hyperparameters	Accuracy *	F1 Score *	MCC *
SVM + RBF	C = 100, kernel = ‘rbf’	99.79	99.80	99.58
SVM	default	98.95	98.99	97.93
Decision Tree	criterion = ‘gini’, max_depth = 4	99.37	99.40	97.93
Bagging Classifier	base_estimator = dt, n_estimators = 300, max_samples = 0.5	99.37	99.40	98.74

* Unit of measurement considered as percentage.

**Table 4:** Estimated performance over Dataset-1 under case-2.

Classifier Used	Best Performance with Hyperparameters	Accuracy *	F1 Score *	MCC *
SVM + RBF	C = 100, kernel = ‘rbf’	80.00	81.08	63.21
SVM	Default	82.85	84.21	67.68
Decision Tree	criterion = ‘gini’, max_depth = 4	85.71	87.17	72.34
Bagging Classifier	base_estimator = dt, n_estimators = 300, max_samples = 0.5	91.42	92.68	82.49

* Unit of measurement considered as percentage.

**Table 5:** Estimated performance over Dataset-2 under case-2.

Classifier Used	Best Performance with Hyperparameters	Accuracy *	F1 Score *	MCC *
SVM + RBF	C = 100, kernel = ‘rbf’	98.33	98.41	98.75
SVM	default	99.37	99.39	98.75
Decision Tree	criterion = ‘gini’, max_depth = 4	99.58	99.60	99.16
Bagging Classifier	base_estimator = dt, n_estimators = 300, max_samples = 0.5	99.37	99.40	98.74

* Unit of measurement considered as percentage.

**Table 6:** Estimated performance over Dataset-1 under case-3.

Classifier Used	Best Performance with Hyperparameters	Accuracy *	F1 Score *	MCC *
SVM + RBF	C = 100, kernel = ‘rbf’	91.42	91.42	83.00
SVM	Default	91.42	90.32	82.78
Decision Tree	Criterion = ‘gini’, max_depth = 4	91.42	92.30	84.01
Bagging Classifier	base_estimator = dt, n_estimators = 300, max_samples = 0.5	94.28	94.73	89.11

* Unit of measurement considered as percentage.

**Table 7:** Estimated performance over Dataset-2 under case-3.

Classifier Used	Best Performance with Hyperparameters	Accuracy *	F1 Score *	MCC *
SVM + RBF	C = 100, kernel = ‘rbf’	99.37	99.38	98.75
SVM	default	100	100	100
Decision Tree	Criterion = ‘gini’, max_depth = 4	99.58	99.56	99.16
Bagging Classifier	base_estimator = dt, n_estimators = 300, max_samples = 0.5	100	100	100

* Unit of measurement considered as percentage.

**Table 8:** Comparison with existing approaches.

Proposed by	Decomposition Methods	Methods for Feature Extraction from Coefficients/IMFs	Feature Concatenation from Decompositions Methods	Datasets	Classifiers	Performance		
						ACC (%)	F1 Score (%)	MCC (%)
Vipin Gupta et al. [4]	FAWT	Cross correntropy, log energy entropy, SURE	No (Single Decomposition method used)	Dataset-2 (single channel)	LS-SVM, KNN	94.41, 93.80	-	89, 88
Anurag Nishad et al. [19]	TQWT	Cross-information potential	No (Single Decomposition method used)	Dataset-2 (single channel)	RF	99	-	-
Mehdi Omidvar et al. [7]	DWT	Standard deviation, mean, band power, Hjorth mobility, Hjorth complexity, Shannon entropy, log-energy entropy, maximum, kurtosis, skewness and median	No (Single Decomposition method used)	Dataset-2 (single channel)	ANN, SVM	100, 100	-	-
Duo Chen et al. [8]	DWT	Max, min, mean, standard deviation, skewness, kurtosis, Energy, normalized standard deviation and normalized energy	No (Single Decomposition method used)	Dataset-1 (multi-channel) And Dataset-2 (single channel)	SVM with RBF kernel	92.30 and 99.33 (overall accuracy over Dataset-1 and Dataset-2, respectively)	-	-
Muhammad Kaleem et al. [10]	EMD	Projection coefficients value (for details refer [10])	No (Single Decomposition method used)	Dataset-1 (multi-channel)	SVM	92.91	-	-
Inung Wijayanto et al. [12]	EMD, coarse-grained (CG)	Fractal Dimension from EMD and CG	No (extracted features individually fed into classifiers)	Dataset-2 (single channel)	KNN, RF and SVM	99, 99 and 100	-	-
Asmat Zahra et al. [13]	MEMD	Instantaneous frequency and amplitude extracted using Hilbert transfor	No (Single Decomposition method used)	Dataset-2 (single channel)	ANN	87.20	-	-
C. Shahnaz et al. [14]	EMD-Wavelet Analysis	DWT applied over IMFs and after that variance, skewness and kurtosis extracted from level 4 DWT coefficients	Partially (but different from our proposed work)	Dataset-2 (single channel)	KNN	100	-	-
Shaik. Jakeer Hussain et al. [15]	DWT and EMD	Mean weighted frequency	No (two ecomposition methods used separately)	Dataset-1 (multi-channel)	ANN	97.18	-	-
Marzhan Bekbalanova et al. [17]	DWT and EMD	Mean, variance, skewness and kurtosis	No (two Decomposition methods used separately)	Dataset-2 (single channel)	SVN, KNN and decision tree	DWT: 99, 97.5, 100 EMD: 100, 100, 96.25	-	-
Proposed	DWT and EMD	Mean, variance, standard deviation, curve length, skewness, kurtosis, minima and rms	DWT coefficient-based feature matrix and EMD IMF-based feature matrix has been concatenated	Dataset-1 (multi-Channel)	SVM, SVM-RBF, decision tree, bagging classifier	91.42, 91.42, 91.42, 94.28	91.42, 90.32, 92.30, 94.73	83.00, 82.78, 84.01, 89.11
				Dataset-2 (single Channel)	SVM, SVM-RBF, decision tree, bagging classifier	99.37, 100, 99.58, 100	99.38, 100, 99.56, 100	98.75, 100, 99.16, 100

## Data Availability

Experimental dataset is publicly available is a https://physionet.org/content/chbmit/1.0.0/ and https://www.upf.edu/web/ntsa/downloads. Source code of this experiment will be available at: https://sites.google.com/site/gcjanahomepage/publications/Publications-Source-Codes.

## Nomenclature

EEG	Electroencephalogram
DWT	Discrete wavelet transform
DCT	Discrete cosine transform
EMD	Empirical mode decomposition
IMF	Intrinsic mode functions
SVM	Support vector machine
RBF	Radial Basis Function
TQWT	Tunable-Q wavelet transforms
FAWT	Flexible analytic wavelet transform
RMS	Root mean square
MCC	Matthews correlation coefficient
AC	Approximate coefficient
DC	Detailed coefficient
SOTA	State of the art
KNN	k-nearest neighbors algorithm
ANN	Artificial neural network
RF	Random Forest Classifier
DT	Decision Tree Classifier
